# Use of intumescent flame-retardant systems in epoxy adhesives for debonding purpose

**DOI:** 10.1016/j.heliyon.2024.e25240

**Published:** 2024-01-28

**Authors:** Oussema Kachouri, Julien Bardon, David Ruch, Abdelghani Laachachi

**Affiliations:** Luxembourg Institute of Science and Technology (LIST), Department of Materials Research and Technology (MRT), Bommelscheuer – 5, ZAE Robert Steichen, L-4940, Hautcharage, Luxembourg

## Abstract

This work investigates the use of intumescent flame-retardant additives as a new debonding solution to disassemble bonded aluminum substrates. Melamine polyphosphate (MPP) or ammonium polyphosphate (APP) was incorporated into an epoxy adhesive joint as both an acid source and a swelling agent with this stimulus responsive behavior being triggered by heating. The ability of the system containing intumescent additives to swell and foam under heat radiation was efficiently exploited to provide enough local pressure to induce porosities and cracks at the interface, facilitating the disassembling of bonded aluminum substrates. Several aluminum/intumescent-epoxy/aluminum laminates were assembled and tested to assess the influence of the MPP and APP content on the mechanical strength of the joints. The structural, morphological, mechanical, and thermal properties of these modified epoxy resins and assemblies with aluminum substrates were studied using Scanning Electron Microscopy (SEM), a pull-off test, Differential Scanning Calorimetry (DSC), and Thermogravimetric Analysis (TGA). The ability of the intumescent-modified joints to support temperature-controlled debonding was evaluated using an oven. The lower debonding temperatures found were comparable to laminates with unmodified epoxy joint systems. Our patented debonding on-demand technology, based on an intumescent flame-retardant system, represents a promising treatment for multi-material structures and will enable products to be recycled at the end of their service life.

## Graphical abstract

**Figure:**
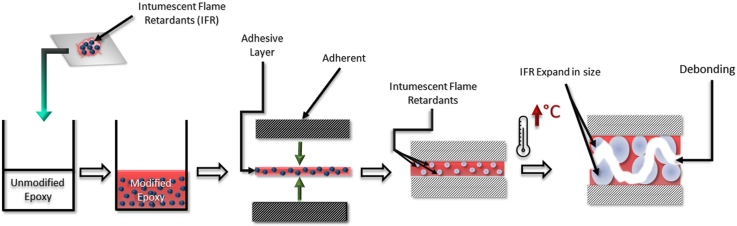

## 1 Introduction

Adhesively bonded joints have profoundly influenced material production methods. The construction of materials has transitioned from mechanical joining methods, such as riveting, screwing, and thermal joining (like welding) to systems that bind multiple materials together by incorporating an adhesive layer into the assembly [1,2]. Owing to its ability to combine and thin dissimilar kinds of materials, adhesive bonding has streamlined the development of innovative, lighter-weight assemblies that boast superior mechanical qualities [3]. This process eliminates the need for the additional components typically used in classical methods, such as screws. Indeed, the adhesive joints exhibit increased resilience to application stresses, thereby conveying superior fatigue resistance and an extended fatigue life [4,5]. The employment of lighter materials in this process addresses a critical environmental concern, since the reduction in the overall weight of the finished products results in a significant in carbon footprint decrease, due to lower energy consumption during both manufacturing and use [6]. Nevertheless, the recyclability of these assemblies, for instance in the automotive industry, remains constrained due to the absence of suitable design and debonding technologies [7]. The recycling processes available at present for reclaiming adherents remain both costly and time-consuming [8].

The imperative for recycling or repair has spurred the evolution of adhesive technologies capable of on-demand debonding in response to specific triggers or stimuli such as heat, electrical current, and chemical reactions. Various proven adhesive methods, including reverse Diels-Alder, electrically induced debonding, and the integration of thermally expanding particles, have previously been investigated, and technical solutions are already accessible [[8], [9], [10]]. Furthermore, certain debonding technologies, such as those based on Diels-Alder [11] or reversible supramolecular methods [[11], [12], [13], [14], [15]], offer reversibility for the debonding process. However, due to the inferior mechanical strength of the corresponding adhesive materials, these technologies are unsuitable for structural bonding. Moreover, these methods present a significant limitation, as they require the specific chemical tuning of the adhesives in advance. Conversely, debonding technology based on electrically induced debonding is initiated by an electrochemical reaction at the interface between the adhesive and anodic adherent [16,17]. While this method is notable for not requiring a heat stimulus, it is only compatible with conductive adherents or requires the addition of metal adherent patches [[16], [17], [18]].

Moreover, promising technologies exist that employ a heat trigger to induce debonding. These technologies involve the incorporation of special particles or agents into the adhesive to enable debonding, including thermally expanding particles [[19], [20], [21], [22]], foaming agents [23], migrating agents [24], and expandable graphite [25].

While these technologies are suitable for structural bonding, their incorporation into the adhesive matrix impairs joint strength and predisposes it to aging [25,26]. This is well-documented in the case of thermally expanding particles in an epoxy adhesive, where the incorporation of 25 % wt. of particles into the epoxy leads to a decrease of 33 % in the lap shear strength of the modified adhesive [22]. Moreover, most of these technologies are incompatible with epoxy thermosets, despite epoxy adhesives being the primary choice for structural adhesive bonding. Although many patents have been granted for different methods of separating bonded joints, few technologies are commercially available [8]. This underscores the need to develop a structural bonding solution that is easy to apply, resistant to aging, applicable to adhesive thermosets, and does not result in the degradation of the joint strength.

The focus of this study is epoxy adhesives, as they are the standard and most effective solution for high-performance structural bonding [27]. Modifications to epoxy resins occur primarily via two methodologies. The first, known as reactive modification, integrates a component during the polymerization phase. However, this approach is fraught with challenges, requiring substantial developmental exertions in chemical synthesis at both the laboratory and industrial scales to develop a product suitable for use [28]. Conversely, the second methodology, the additive approach, has proven to be more versatile and easier to employ, and is highly appealing to both the industrial and academic sectors [29], due to the straightforwardness of mixing additives into the epoxy adhesive prior to the curing process.

Among the functional additives that can be incorporated into an epoxy material, flame retardants are often utilized for specialized applications. Flame retardants are a chemical compound that can be applied to a multitude of manufactured materials, including plastics, textiles, and coatings. These compounds are activated in the presence of a flame source and are designed to prevent or delay the spread of fire via various physical and chemical mechanisms [30,31]. Flame retardants commonly feature halogens, phosphorus, nitrogen-based chemicals, and mineral fillers. Among the various flame-retardant technologies explored, intumescent systems have emerged as the most promising. These systems rely on a polymer's ability to expand or foam upon exposure to heat, thereby creating a porous layer that acts as a barrier against heat or gas transfer. Typically, an intumescent system consists primarily of an acid source like APP that, when heated, discharges polyphosphoric acid. This, in combination with a swelling agent such as melamine, facilitates expansion through the release of inert gases (NH_3_, CO_2_, H_2_O). This sequence culminates with a carbon source, for instance, pentaerythritol, forming a cross-linked char layer. The most frequently documented intumescent system is APP/pentaerythritol, the mechanism of which has been comprehensively elucidated in the work of Schartel and coworkers [32]. Indeed, as noted earlier, intumescent flame retardants have traditionally been used in fire-proofing applications [33]. However, this study aims to innovatively utilize the foaming and swelling effects of these systems, as well as their early thermal degradation. Upon heating, the epoxy layer bridging the two substrates expands, forming a thermally degraded porous layer that diminishes its mechanical properties, thereby facilitating a debonding effect.

This study focuses on the innovative technology patented by our research team, in which an epoxy joint is debonded using flame retardant agents activated by heat [34]. The behavior of two well-known epoxy adhesive systems (DGEBA/DDS and DEGBA/DETA), which have been modified by the inclusion of traditional intumescent flame retardants (APP and MPP) at various filling rates, will be examined. In particular, the degradation of the joint strength as a function of thermal loading and adhesive modification will be studied. Furthermore, the thermochemical characteristics of the modified adhesives will be investigated to provide complementary information about their thermal behavior.

Ultimately, the objective of the present study is not to utilize intumescent flame retardants in their traditional capacity to improve the flame retardancy of materials, but rather to leverage these materials to pioneer an innovative and effective debonding strategy that is specifically tailored to thermosets.

## 2 Materials and methods

### 2.1 Adhesives

Bisphenol A diglycidyl ether (D.E.R.332, commonly abbreviated as BADGE or DGEBA), was selected as a low common viscosity epoxy resin. Two distinct hardeners were used, an aromatic high-crosslinking agent, 4-Aminophenyl sulfone (abbreviated as DDS), and an aliphatic flexible hardener, Diethylenetriamine (abbreviated as DETA). All products were supplied by Sigma-Aldrich Germany.

### 2.2 Intumescent flame-retardant systems

The intumescent flame-retardant systems selected for this study were melamine polyphosphate (MPP) and Ammonium polyphosphate (APP), supplied by CIBA Germany. The commercial name of MPP is MELAPUR 200.

Table 1 summarizes the main characteristics of the flame retardants with information supplied by the manufacturer.

**Table 1:** Technical characteristics of the flame retardants used in this study.

Flame retardant	MPP (MELAPUR 200)	APP (Exfolit AP 422)
Nitrogen Content (wt.%)	42–44	14–15
Phosphor Content (wt.%)	12–14	31–32
Particle Size (D98) (μm)	70	–
Particle Size (D50) (μm)	–	17
Real Density (g/cm³)	1.85	1.9

### 2.3 Preparation of the formulations

**Fig. 1:**
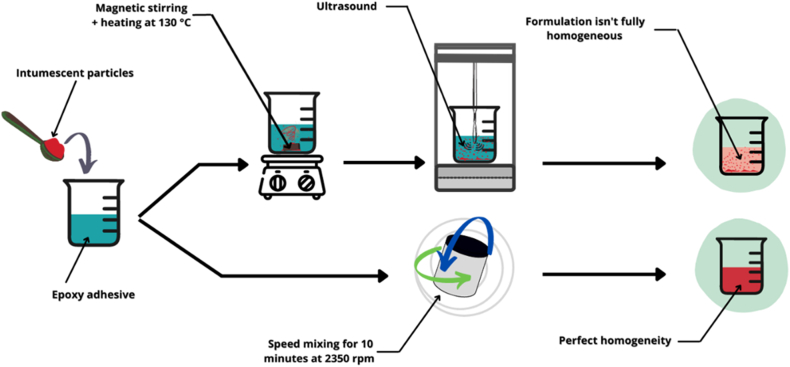
Scheme of the experimental approach for formulation preparation.

As illustrated in Fig. 1, two different experimental paths were explored. The first was accomplished by mixing the additives into the epoxy after heating it to 130 °C to reduce its viscosity. After magnetic stirring for approximately 15 min at a speed of 300 rpm, the formulation was mixed with an ultrasonic probe (Hielscher Ultrasonic Processor UP400) for about 15 min to improve its dispersion into the DGEBA matrix.

In the alternative approach, the mixtures were blended for 10 min at 2350 rpm with a Hauschild speed mixer (Speed Mixer DAC 400) to guarantee that the formulations were free of bubbles and homogeneous. The hardener was added at the same time as the additives for the DGEBA/DDS systems, but was not used for the DGEBA/DETA systems in order to avoid any crosslinking before the crosslinking step.

The use of a speed mixer is more efficient than utilizing magnetic stirring and an ultrasonic probe for dispersion, as this requires a longer preparation time and results in a phase separation between the epoxy and the additives after a few hours.

### 2.4 Curing cycles

A two-step oven-curing cycle was adopted for the DEGBA/DDS samples, which were then cured at 180 °C for 2 h followed by a 2-h post-cure at 220 °C.

For the DEGBA/DETA system, samples were cured at room temperature for 24 h, followed by a 2-h post-cure at 100 °C.

### 2.5 Loading rates

To provide comprehensive insights into our investigation, loading rates of 10 wt%, 20 wt%, and 40 wt% were selected. These specific rates were chosen to span a wide range of loading conditions, with 10 wt% representing a low loading rate and 20 wt% serving as an intermediate loading rate. Throughout our data collection, the 20 wt% consistently yielded the best results in terms of joint strength and debonding. Consequently, it was decided to double this loading rate to 40 wt%, characterizing it as a high loading rate, to explore the loading limit and its impact on various adhesive properties.

It is worth noting that at this high loading rate of 40 wt%, a degradation in the processability of the adhesive was observed, highlighting a critical threshold that affects the preservation of adhesive integrity within the context of our experimental conditions.

### 2.6 Assembly preparation

#### 2.6.1 Adherents

In order to conduct mechanical testing and debonding experiments, 5 mm-thick aluminum substrates were selected and different plate dimensions were employed. For the mechanical screening, 9 cm × 9 cm substrates were selected that could accommodate 9 pull-off joints. For the debonding test, 4 cm × 4 cm substrates were chosen that supported only one single junction. All joints consisted of an aluminum plate on one side and a pull-off dolly with a 20 mm radius supplied by PosiTest AT on the other.

#### 2.6.2 Specimen design

Several steps were required to prepare the surface of the joints to guarantee a uniform treatment that was as repeatable as possible. This specific surface treatment was chosen after extensive experimentation with a variety of factors, including solvents and treatments such as substrate heating and plasma treatment.

**Fig. 2:**
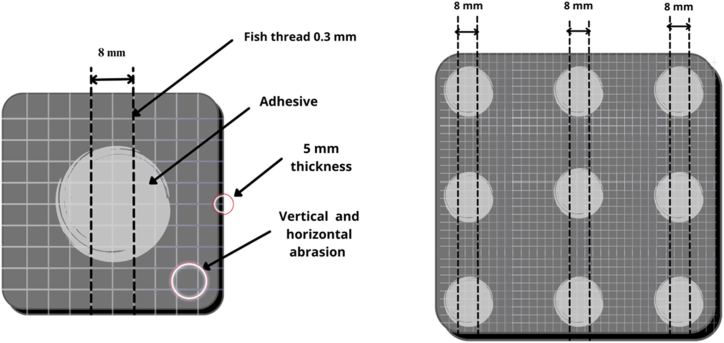
Design and preparation scheme of the adherents.

First, all aluminum specimens were cleaned with ethanol. Then, using SiC FEPA 80 sandpaper supplied by Struers United Kingdom, all metallic plates were abraded to a smooth finish via vertical and horizontal grinding. They were subsequently cleaned with ethanol again to remove abrasion particles. Epoxy adhesive was deposited manually between two 0.8 nylon fishing threads and spread by applying pressure with the aluminum dolly. The threads ensured that the joint thickness had a reproducible value (300 μm), regardless of the pressure applied by the operator and the viscosity of the modified adhesive. Fig. 2 highlights the different manufacturing details for both types of specimens. The joined dolly was taped to the aluminum substrates to maintain the joint geometry during the curing cycle.

All these preparation steps were carefully evaluated in order to (i) increase the joint strength by increasing the aluminum-epoxy adhesion and (ii) increase the joint strength reproducibility, thereby increasing the possibility of discerning the effects of the epoxy modification.

#### 2.6.3 Bulk resin

Plates of bulk resin were formed by pressing the resin into a steel mold during curing to ensure the removal of all air bubbles. A thin layer of release agent was pre-deposited into the mold to ensure that the resin plate could be removed easily after curing.

Both unmodified and modified samples, as well as the adhesive joints for both systems, were manufactured in the same manner. These plates were then removed from the mold and the samples required for DSC and TGA were cut from them.

### 2.7 Testing methods

#### 2.7.1 Joint strength

A comparative pull-off testing approach was used to evaluate the mechanical characteristics of each different adhesive formulation. Every modified adhesive from every hardener was compared to the corresponding unmodified reference, and all the modified adhesives were compared to one another in order to evaluate the impact of increasing the weight percentage of additives in the formulation. This was done using a pull-off tester provided by DeFelsko (PosiTest AT-A). A total of six joints were examined to determine the standard deviation of the results, and for each formulation series, a dedicated reference (non-modified) was also tested.

#### 2.7.2 Debonding test

**Fig. 3:**
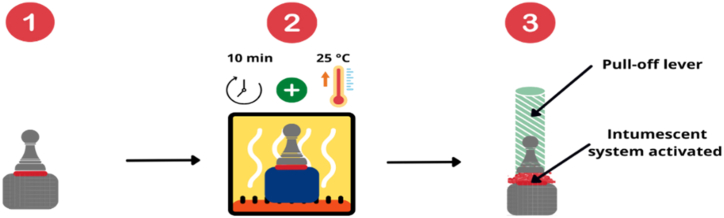
Schematic representation of the debonding test.

A modified version of the pull-off test was developed to assess the impact of our additives on the joint strength at high temperatures. To do this, as illustrated in Fig. 3, the joints were subjected to a temperature at incremental gradients for 10 min at a time. The evaluation starting temperature was 5 °C above the curing temperature, and increased in increments of 25 °C until the reference adhesive (non-modified) was severely degraded, at around 400 °C for the DEGBA/DDS adhesive. It was possible to monitor the thermo-mechanical degradation of the adhesive joints in a reproducible way. The transition temperature from the temperature range in which the joints exhibited a high mechanical performance and the temperature range in which the joint strength was low allowed us to determine a debonding activation temperature.

This debonding temperature is assumed to be the temperature at which the joint stress at failure is 10 % of the maximum stress recorded for a given formulation. This assumption is discussed a posteriori in the Discussion section. Since data points give the discrete values of joint stress, the temperature corresponding to the value at 10 % of the maximum stress was calculated by linear interpolation. Three tests were performed at all data points in order to evaluate the standard deviation of the procedure.

#### 2.7.3 Thermogravimetric analysis (TGA)

An analysis of the influence of the additives on the degradation temperature of the epoxy resin adhesive system was carried out in this work using a TGA 2 SF apparatus from Mettler Toledo, United States. The testing conditions were inert gas, and air under a gas flow of 100 cm^3^ min^−1^ with alumina crucibles (70 μl) containing 15–18 mg of sample. The temperatures ranged from room temperature to 900 °C. The run was carried out in dynamic conditions at a constant heating rate of 10 °C min^−1^. For each formulation, the onset temperature corresponding to a 5 % mass loss was recorded.

#### 2.7.4 Differential scanning calorimetry (DSC)

The glass transition temperatures (Tg) were measured with a DSC3+ instrument with a heating rate of 10 °C min^−1^ from Mettler Toledo, United States. The crosslinked samples were placed into aluminum crucibles.

#### 2.7.5 Scanning electron microscopy (SEM)

In order to investigate the dispersion of the additives into the epoxy resin, modified and unmodified epoxy resins were assembled between two substrates of aluminum in a simple recovery joint. A 300 μm-thick fish thread was used to regulate the thickness of the joint between the substrates. The cross-sections of these assemblies were mirror-polished. These materials were investigated with a FEI QUANTA FEG 200 environmental scanning electron microscope (ESEM).

## 3 Results

This section elucidates the results of the experiments outlined above by investigating the influence of APP and MPP additives on the joint properties, including joint strength, debonding temperatures, failure modes, physicochemical properties, and thermal properties. In addition, the failure modes and dispersion of the fillers in the adhesive were closely examined.

### 3.1 Joint strength

**Fig. 4:**
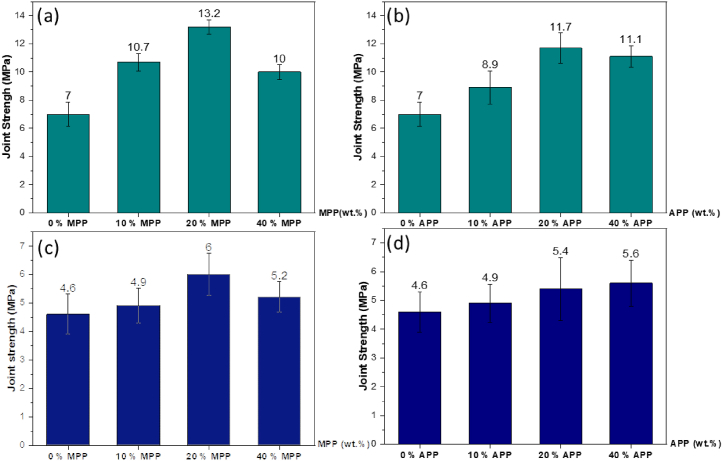
Joint strength of unmodified and modified resin epoxy at room temperature: (a) DGEBA/DDS with MPP, (b) DGEBA/DDS with APP, (c) DGEBA/DETA with MPP, (d) DGEBA/DETA with APP.

The adhesive properties of epoxy formulations were evaluated using “dolly-plate” assemblies. Fig. 4 depicts the evolution of the adhesive strength at room temperature for an epoxy with varying amounts of MPP or APP.

It was observed that the introduction of MPP and APP as additives in epoxy resins substantially enhanced the adhesive joint strength compared to the unmodified epoxy reference: MPP at 20 wt% and APP at 20 wt%, with the MPP strength increasing from 7 to 13.2 MPa, and APP strength from 7 to 11.7 MPa, respectively. For DGEBA/DETA systems (with incorporated MPP and APP), the increase in joint strength was moderate and potentially insignificant considering the values of standard deviation. For instance, joint strength rose from 4.6 MPa to 6 MPa with the presence of 20 wt% MPP. Although the findings were more significant in the DGEBA/DDS adhesive matrix than in the DGEBA/DETA matrix, no decline in joint strength was observed in any formulation. Given that all joint failures were adhesive, and that joint strength increased after a modification of the adhesive, these results suggest that the presence of both APP and MPP additives did not adversely impact adhesion at the adhesive/substrate interface, and is therefore a notable outcome. Furthermore, the values of joint strength measured by pull-off testing in the present study are of similar magnitude to those from a previous study in which bio-based epoxy adhesives were bonded to aluminum surfaces [35]. These joint strength values were in the range of 4–6.5 MPa.

It is also worth highlighting the positive impact of incorporating APP or MPP. Fig. 4 illustrates that the joint strength at 20 wt% of the additives surpassed that at 10 wt% in all cases. However, at 40 wt%, the values dropped below those at 20 wt%. This decline may be associated with the observations made during the execution of the experimental protocol. When the mass fraction of additives exceeded 20 %, the adhesive became excessively viscous, which compromised the joint quality. Another potential explanation for the decrease in joint strength could be the higher presence of aggregates when loading surpasses 20 %, which might cause early joint failure. Based on the joint strength assessment (Fig. 4), it is plausible to conclude that joint strength peaks at 20 wt% of additive concentration. As observed later (section 3.3), when performing joint testing, there is a failure in the adhesive at the aluminum substrate – epoxy joint interface. Furthermore, the addition of stiff microparticles, such as MPP or APP, to an epoxy matrix generally leads to a significant improvement of the composite elastic modulus, as observed for the epoxy with an APP filler [36]. It is therefore hypothesized for the present study that the addition of polyphosphate particles to the epoxy joint leads to an increase in the joint elastic modulus, which reduces the elastic modulus mismatch at the epoxy – aluminum interface. This in turn delays the failure at the interface for a normal given stress and could lead to an increase in joint strength. When the loading rate exceeds 20 %, this reinforcement mechanism is still valid, but the formation of particle agglomerates could both reduce the joint elastic modulus by creating additional porosity and trigger the premature failure of the joint, starting within the agglomerate and possibly propagating to the epoxy aluminum interface.

### 3.2 Debonding test

To investigate the thermal response of the intumescent compositions within the epoxy adhesive on the disassembly of the aluminum/epoxy/aluminum assembly, a debonding test was conducted using a pull-off method after heat treatment, as detailed in the experimental section.

**Fig. 5:**
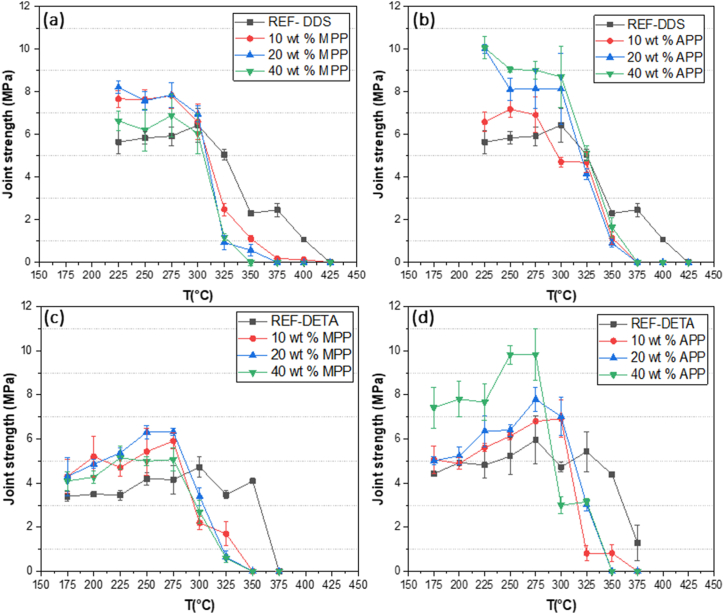
The evolution of the adhesive strength as a function of the temperature of aluminum/epoxy/aluminum assemblies: (a) DGEBA/DDS with MPP, (b) DGEBA/DDS with APP, (c) DGEBA/DETA with MPP, (d) DGEBA/DETA with APP.

Fig. 5 presents the evolution of adhesive strength as a function of temperature for aluminum/epoxy/aluminum assemblies containing different amounts of MPP or APP. The thermal response of every adhesive composition with both hardener systems was analyzed in incremental temperature steps until the reference epoxy in each system was completely thermally degraded. As displayed in Fig. 5, DGEBA/DDS systems consistently exhibited stronger joint strength than the DGEBA/DETA systems. As anticipated, non-modified DGEBA/DDS formulations proved more resilient to higher temperatures, with DGEBA/DDS joints retaining some mechanical resistance beyond 400 °C, while DGEBA/DETA joints fully degraded after reaching 375 °C.

Throughout the application range (beginning at 225 °C for DGEBA/DDS and 175 °C for DGEBA/DETA and ending at the debonding temperature), modified adhesives with MPP and APP maintained their mechanical properties effectively. Across all loading rates (10 %, 20 %, 40 %) for modified formulations, the mechanical strength exceeded that of the reference adhesive. Additionally, all formulations with APP and MPP exhibited a slight increase in joint strength values when subjected to high temperatures. Two theories have been developed to explain these findings: firstly, the curing and post-curing cycles may not have achieved a full crosslinking of the network, and secondly, the addition of flame-retardant additives may have altered the physicochemical properties of the epoxies. Further investigations of this latter hypothesis were conducted through DSC experiments.

The adhesive formulations containing 20 wt% and 40 wt% of additives exhibited the highest strength during heating. As can be seen in Figs. 5 (a), 20 wt% MPP outperformed 40 wt% MPP, with the former peaking at a maximum value of 8.2 MPa while the latter did not exceed 6.4 MPa. However, in Fig. 5 (b), at 225 °C, both 20 wt% APP and 40 wt% APP show comparable values, but as the temperature rose, the more concentrated formulations demonstrated superior strength until the debonding temperature was reached. This observation can be rationalized by considering the particle size of our additives and their dispersion within the DGEBA matrix. Given the much smaller particle size of APP powders, better dispersion is expected.

In the presence of the MPP additive, as demonstrated in Fig. 5 (a) and (c), the mechanical performance of the joints abruptly deteriorated at 325 °C, falling from 7 MPa in both DGEBA/DDS and DGEBA/DETA at 300 °C to less than 1 MPa for both systems at 325 °C. Conversely, for formulations containing APP additive, as shown in Fig. 5 (b) and (d), debonding occurred after 350 °C, as the joint strength decreased from 8.1 MPa in 20 wt% APP for DGEBA/DDS and 6.4 MPa for the same mass fraction for DGEBA/DETA to less than 0.5 MPa. The debonding temperatures (T_°deb_) of the formulations under study were extracted from Fig. 5 and are presented in Table 1.

The inclusion of APP or MPP in the epoxy resin significantly lowered the debonding temperature of the resin, with temperature reductions ranging from 17 °C to 86 °C. Notably, the greatest decrease was achieved with the inclusion of 20 wt% MPP.

### 3.3 Examination of the failure modes

**Fig. 6:**
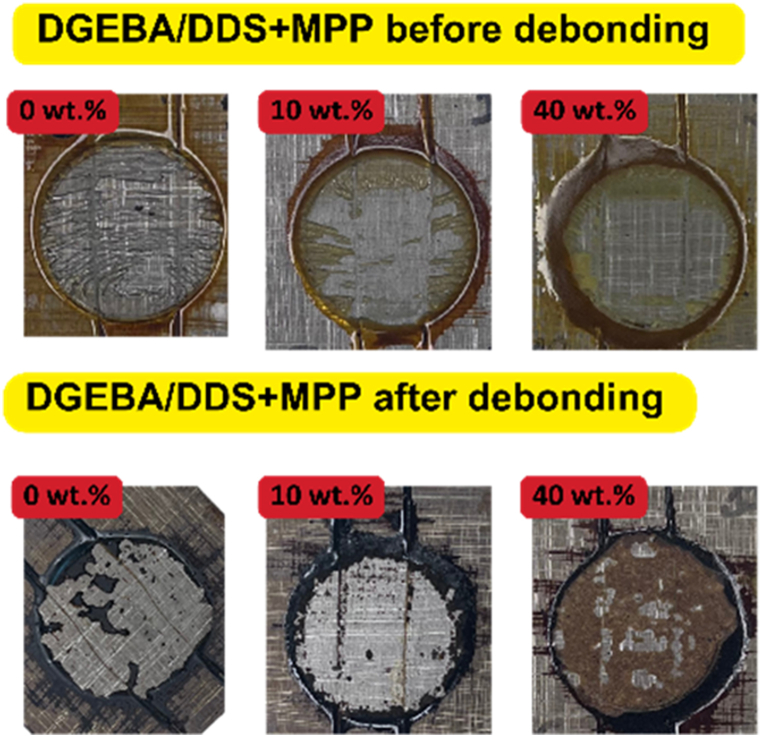
Sample observation of failure modes.

Examination of joint failures was conducted on samples collected at ambient temperatures and those obtained at the debonding temperature (Fig. 6). These failure modes were visually evaluated and two primary types were identified: mixed failure and cohesive failure. It was observed that failure modes transitioned from cohesive to mixed failures as the loading of flame retardants in the adhesives increased. Additionally, adhesive failure was observed, particularly in the non-modified adhesive systems, in the post-debonding phase.

### 3.4 Influence of the additives on the physicochemical properties

#### 3.4.1 Influence on the glass transition temperature

To investigate the impact of additives and filling rates on the resin matrix, Differential Scanning Calorimetry (DSC) measurements were taken for all formulations at different loading rates and for both hardeners. The results obtained for glass transition temperature (Tg) are summarized in Table 3.

**Table 2:** Debonding temperatures (T°deb) of unmodified and modified resin epoxy with APP and MPP additives.

	T_°deb_, in DGEBA/DDS (°C)	T_°deb_, in DGEBA/DETA (°C)
Ref. (neat resin)	411	372
10 % APP	360	355
20 % APP	352	344
40 % APP	361	342
10 % MPP	358	341
20 % MPP	325	326
40 % MPP	325	329

**Table 3:** Temperatures of the glass transitions of the formulations studied in DGEBA/DDS and DGEBA/DETA.

	Tg in DEGBA/DDS (°C)	Tg in DEGBA/DETA (°C)
Ref.	206	155
10 % MPP	206	155
20 % MPP	206	155
40 % MPP	207	155
10 % APP	208	155
20 % APP	208	156
40 % APP	208	156

These results show that the incorporation of MPP and APP additives, at various mass fractions, exerts a negligible influence on the glass transition temperature (Tg) of the epoxy matrices. As illustrated in Table 2, the Tg of the reference DGEBA/DDS system registered at 206 °C, whereas when MPP was introduced at a 40 % weight ratio, the Tg exhibited a minor deviation of 1 °C, reaching 207 °C. The same behavior was exhibited in all other formulations.

#### 3.4.2 Thermogravimetric study

**Fig. 7:**
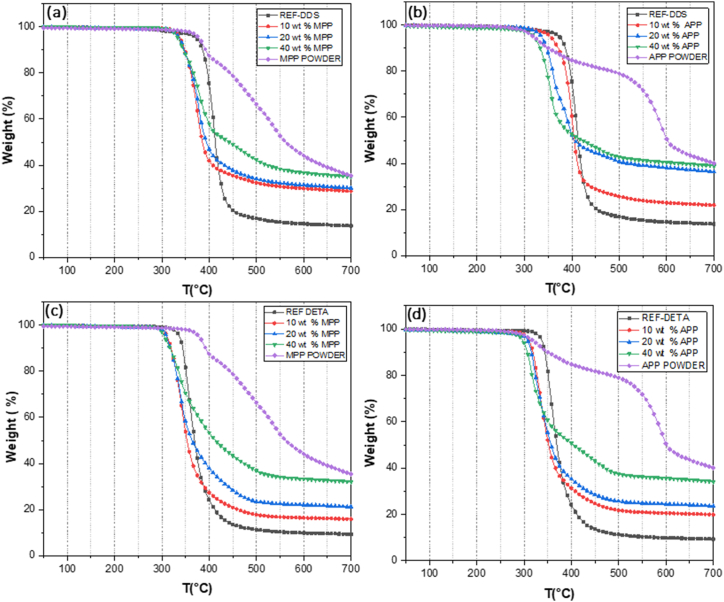
TGA curves of all formulations under N2, (a) DGEBA/DDS with MPP, (b) DGEBA/DDS with APP, (c) DGEBA/DETA with MPP, (d) DGEBA/DETA with APP.

The thermal degradation of modified DGEBA epoxies was assessed in comparison to that of unmodified DGEBA epoxies. Fig. 7 presents the TG curves for DGEBA-DDS and DGEBA-DETA with varying contents of MPP and APP (10, 20, and 40 wt%). The onset temperatures of degradation at 5 % of weight loss (T°_onset5 %_) are reported for each formulation in Table 4.

**Table 4:** Onset temperatures of degradation at 5 % of weight loss.

	T°_onset5 %,_ in DGEBA/DDS (°C)	T°_onset5 %,_ in DGEBA/DETA (°C)
Ref. (neat resin)	373	338
10 % APP	355	311
20 % APP	336	308
40 % APP	320	297
10 % MPP	336	311
20 % MPP	335	310
40 % MPP	334	305

The TGA results indicate that the inclusion of either MPP or APP in the DGEBA resins reduced their thermal stabilities. In fact, with the APP additive (Fig. 7 (b) and (d)), T°_onset5 %_ decreased as the mass fraction of APP increased. The most significant decrease in T°_onset5 %_ was observed with the incorporation of APP at a 40 % loading rate for both resins, DGEBA/DDS and DGEBA/DETA. The lowest onset temperature (297 °C) was noted for APP at a 40 % loading rate for DGEBA/DETA, while the greatest decrease in onset temperature (53 °C) was observed for APP at a 40 % loading rate for DGEBA/DDS.

For MPP additives (Fig. 7 (a) and (c)), it was observed that the T°_onset5 %_ remained relatively consistent, regardless of the mass fraction. In DGEBA/DDS, the T°_onset5 %_ for 10 %, 20 %, and 40 % MPP was approximately 334 ± 2 °C. Conversely, increasing the mass fraction affected only the residue amount at 700 °C.

Degradation always commences earlier in DGEBA/DETA than in DGEBA/DDS systems. Thus, the deterioration of thermal properties in adhesives is dependent both on the type of flame retardant used and its properties, as well as the matrix into which the flame retardant is introduced.

**Fig. 8:**
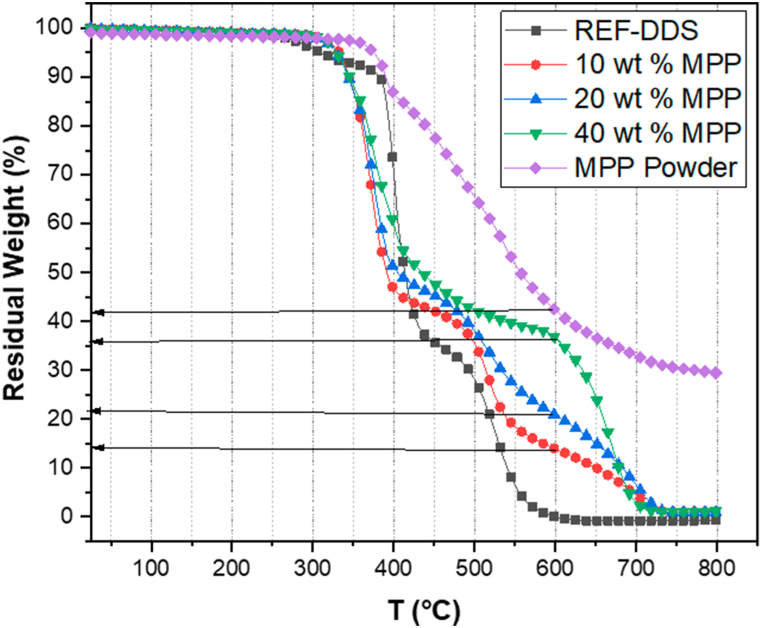
TGA curves of different DGEBA-DDS based formulations with MPP under AIR.

Regarding the mechanism or mode of action, the phosphoric acid released by MPP or APP is expected to react with the carbon source (the DGEBA matrix in our case), resulting in a higher formation of char. In our study, the TGA curves under air were utilized to verify the quantity of residue and its evolution with the addition of additives (Fig. 8). Table 5 details the amount of residue from the different formulations at 600 °C. The residue amount obtained experimentally was compared with the residue calculated from the theoretical curve according to the mixing law. The deviation between the theoretical and experimental data indicates the presence of an interaction, facilitated by the activation of the intumescent systems.

**Table 5:** Theoretical and experimental mass residue of the different flame retardants wt.% within both DGEBA matrixes at 600 °C.

Flame retardants	Theoretical residue in %	Experimental value (DGEBA/DDS) in%	Experimental value (DGEBA/DETA) in %
10 % MPP	4.2	14.015	8.9
20 % MPP	8.41	21.17	17.59
40 % MPP	16.82	37.05	34.15
10 % APP	4.98	20.73	14.61
20 % APP	9.96	38.19	30.47
40 % APP	19.92	43.24	37.36

For instance, with 40 wt% of APP, the residue amounts were 38.19 % in DGEBA/DDS and 37.36 % in DGEBA/DETA, while the theoretical value was only 19.92 %. In all DGEBA/DDS systems, the mass residue consistently surpassed the mass residue in DGEBA/DETA systems. This difference could be attributed to the distinct chemical and structural compositions of the hardeners, given that DDS includes more aromatic structures than DETA, which in turn contributes to an increase in the char residue formed during thermal decomposition. Notably, there was less residue when using MPP than when using APP. For example, 20 wt% MPP in DGEBA/DDS resulted in 21.17 % char, whereas 20 wt% APP generated 38.19 %. This is likely due to the presence of melamine molecules in MPP, which contribute to the creation of additional gases that ultimately evaporate as the matrix degrades.

### 3.5 Influence of the dispersion of the additive

**Fig. 9:**
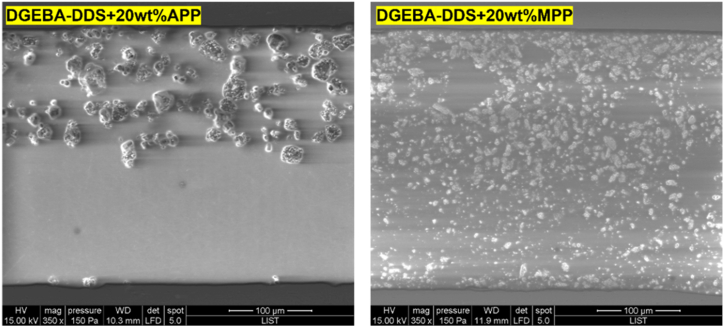
SEM images of the different additives at 20 wt% in DGEBA/DDS, (a) 20 % APP, (b) 20 % MPP, (c) close-up of the epoxy adherend interface.

To elucidate the dispersion characteristics of the additives within the epoxy resin matrix, samples with both modified and unmodified epoxy resins were manufactured between two aluminum substrates, employing a simplistic recovery joint. Fig. 9 presents representative SEM micrographs of both adhesive systems, each containing 20 wt% of MPP and APP.

The images indicate that the MPP and APP additives do not exhibit the same dispersion behavior in the epoxy resin: while MPP additives show well-distributed particles, there is a tendency towards aggregation in the case of APP. Concurrently, a precipitation phenomenon is particularly noticeable with APP, whereby a higher density of particles is observed near the lower aluminum substrate. This trend is amplified at higher loading rates; the primary distinction lies in the significantly escalated levels of agglomeration and precipitation for APP samples. In contrast, the MPP samples maintain a largely homogeneous dispersion despite the visible increase in agglomerate formation.

Furthermore, the specific type of hardener employed does not appear to significantly influence these dispersion states, suggesting the primacy of the additives themselves in shaping the distribution and behavior of the components within the epoxy resin matrix.

## 4 Discussion

Previous works on the debonding effect obtained by the incorporation of a dedicated functional additive into a structural adhesive have studied chemical and physical foaming agents [23], thermally expandable particles [[19], [20], [21], [22]], expandable graphite [25], and migrating agents [24]. To the best of our knowledge, this is the first research that investigates the use of intumescent flame retardants as additives to impart a debonding effect on adhesives.

A methodology was developed in order to determine a debonding temperature. This allowed a direct comparison of the effects on the debonding temperature obtained with the incorporation of various flame-retardant additives at different loading rates. After a statistical evaluation of the correlation between debonding data and thermogravimetry data, the debonding temperature evaluation criterion was selected as 20 % of the maximum load. More precisely, debonding test data and TGA curves were carefully analyzed, and characteristic figures were extracted from the data. For TGA curves, the following temperatures were evaluated:

- The temperature at which 5 % of the initial weight is lost. This temperature is sometimes described as the onset of degradation and is therefore called: T_onset5 %_
- The temperature at which 10 % of the initial weight is lost. This temperature is sometimes described as the onset of degradation and is therefore called: T_onset10 %_
- The temperature at which 20 % of the initial weight is lost, T_weight20 %_
- The temperature at which 50 % of the weight is lost, T_weight50 %_

For the debonding test data, the following temperatures were evaluated:

- The temperature at which 50 % of the maximum joint strength is obtained, T_50 %stress_
- The temperature at which 20 % of the maximum joint strength is obtained, T_20 %stress_
- The temperature at which 10 % of the maximum joint strength is obtained, T_10 %stress_
- The temperature at which the joint strength falls to zero, T_0stress_

After acquiring these datasets for all reference and modified resins, the correlation between data from TGA and debonding tests were evaluated by calculating the Pearson correlation coefficient. The calculated coefficients are presented in Table 6.

**Table 6:** Correlation coefficient values between the datasets (data for all resins).

	T_50 %stress_	T_20 %stress_	T_10 %stress_	T_0stress_
**T**_**onset5** **%**_	0.71	0.71	0.73	0.74
**T**_**onset10 %**_	0.68	0.69	0.71	0.72
**T**_**weight20 %**_	0.59	0.59	0.61	0.66
**T**_**weight50 %**_	0.11	0.16	0.12	0.13

The best correlation between TGA data and debonding tests is observed for T_onset5 %_ and T_0stress_. However, the information contained in T_0stress_, i.e. the temperature at which the joint fails, is of little use since the testing was undertaken at 25 °C, resulting in many modified resins exhibiting the same T_0stress_, which provided little differentiation. Therefore, T_10 %stress_ is preferred as a characteristic debonding value; justifying a posteriori why this particular resin characteristic temperature was selected in the Method section.

The value of correlation coefficient between these two datasets, T_onset5 %_ and T_10 %stress_, shows a rather good, but not perfect, correlation. It is therefore assumed that the phenomena leading to the joint thermal degradation and weight loss are different to those leading to the thermal degradation and mechanical strength of the joint. Nevertheless, observing the TGA results might give a first insight into the capacity of additives to lead to debonding when increasing temperature.

Furthermore, the comparison between debonding data and TGA data, in particular, as shown in Table 5, should be further analyzed. For instance, the onset temperature for TGA tests tends to decrease more for APP incorporation, in particular for 40 % wt.% of APP, than for MPP incorporation. This is not correlated to the debonding data, in which MPP incorporation is more effective than APP, i.e., the debonding temperature is much lower in the case of MPP incorporation.

Observations of SEM pictures showing APP and MPP incorporation show that APP particles are quite big after being incorporated into the epoxy, and that agglomeration certainly occurs. This reduces the contact area between APP and the epoxy matrix, in contrast to MPP where the particles are much smaller, thus increasing the corresponding area of contact with the matrix. It can therefore be assumed that at a given temperature, the intumescence phenomena are enhanced for MPP compared to APP, which leads to better debonding. Lastly, this might explain a lower debonding temperature for MPP.

In addition, it was confirmed that the incorporation of flame retardants had no effect on the physicochemical characteristics of the adhesives, since the Tg remained practically the same after APP or MPP incorporation. This is interesting, since Tg is a critical characteristic of polymers and adhesives, and the addition of these two fillers does not modify this characteristic despite the high loading rate (40 % wt.%) tested in this study.

To the best of the authors’ knowledge, this study is the first to describe how the addition of intumescent flame-retardant additives to an adhesive can lead to a better debonding of the adhesive joint. For this reason, a patent describing this innovative approach was granted [34]. A complete methodology was developed to characterize the thermal and debonding properties of modified adhesive joints. The current debonding temperatures are in the 325–350 °C range after adhesive modification. This is rather high compared to typical debonding temperatures for epoxy adhesives loaded with thermally expandable particles [[19], [20], [21], [22]] or expandable graphite [25] where debonding temperatures are close to 140 °C or 235 °C, respectively. Although the glass transition temperature of the epoxy adhesives and the debonding methods are different for the other debonding approaches, the current debonding temperatures are quite high and further investigations with other intumescent systems are necessary in order to achieve lower debonding temperatures. For instance, the combination of several flame-retardant additives could be investigated to hopefully obtain a synergistic effect between them [37].

Finally, it is also anticipated that the range of applicability of the current study, which was first developed on aluminum-epoxy-aluminum joints, will be extended. More generally, the addition of functional additives in epoxy adhesives to obtain a debonding on-demand function could first be employed to trigger the debonding of fiber-reinforced polymer (FRP) composites, in particular, when the polymer matrix is an epoxy thermoset. Furthermore, a more advanced application could separate reinforcement fibers and matrix in FRPs to allow higher-value fibers to be recycled.

## 5 Conclusion

In this study, a novel methodology was developed to facilitate the easy debonding of adhesive joints, by means of traditional intumescent flame-retardant systems. The incorporation of ammonium polyphosphate and melamine polyphosphate was tested for this purpose and a reduction in debonding temperatures was observed. Our findings showed that these additives did not impact the adhesive joint properties, namely, adhesion and mechanical strength. Furthermore, MPP and APP considerably enhanced the adhesive joint strength compared to unmodified epoxy. To investigate the thermal response of intumescent additives within the epoxy adhesive during the disassembly of the aluminum/epoxy/aluminum assembly, a debonding test was conducted using the pull-off method after heat treatment. In this study, it is shown for the first time that adding intumescent agents such as APP or MPP to an epoxy-based joint can promote the easier debonding of the joint by accelerated thermal degradation of the modified epoxy. The incorporation of flame-retardant agents results in the debonding temperature being reduced by less than 100 °C. Further investigations will be conducted with the introduction of other flame-retardants as debonding agents, in order to activate debonding at even lower temperatures in epoxy-based materials, thereby broadening the range of potential applications of this debonding on-demand technology.

## Additional information

No additional information is available for this paper.

## Data availability statement

The raw/processed data required to reproduce these findings cannot be shared at this time as the data also forms part of an ongoing study.

## CRediT authorship contribution statement

**Oussema Kachouri:** Writing – original draft, Investigation, Conceptualization. **Julien Bardon:** Writing – review & editing, Supervision, Methodology, Investigation. **David Ruch:** Project administration. **Abdelghani Laachachi:** Writing – review & editing, Validation, Supervision, Investigation, Funding acquisition, Conceptualization.

## Declaration of competing interest

The authors declare the following financial interests/personal relationships which may be considered as potential competing interests:Abdelghani laachachi reports financial support was provided by The Luxembourg National Research Fund. Abdelghani laachachi has patent #Multi-Material Disassembly. WO Patent WO2021219736 licensed to licensee.
